# Evaluation of the Computer-Based Intervention Program *Stayingfit* Brazil to Promote Healthy Eating Habits: The Results from a School Cluster-Randomized Controlled Trial

**DOI:** 10.3390/ijerph16101674

**Published:** 2019-05-14

**Authors:** Karine Brito Beck da Silva, Naiá Ortelan, Sheila Giardini Murta, Isabel Sartori, Ricardo David Couto, Rosemeire Leovigildo Fiaccone, Maurício Lima Barreto, Megan Jones Bell, Craig Barr Taylor, Rita de Cássia Ribeiro-Silva

**Affiliations:** 1 Departamento de Ciências da Nutrição, Escola de Nutrição, Universidade Federal da Bahia, Av. Araújo Pinho, 32, Canela, Salvador, BA 40.110-150, Brazil; ritaribeiroufba@gmail.com; 2 Cidacs—Centro de Integração de Dados e Conhecimentos para Saúde, Instituto Gonçalo Moniz, Fundação Osvaldo Cruz, Ministério da Saúde, Parque Tecnológico da Bahia, Rua Mundo, 121, Trobogy, Salvador, BA 41745-7115, Brazil; nana.ortelan@gmail.com (N.O.); fiaccone@ufba.br (R.L.F.); mauricio@ufba.br (M.L.B.); 3 Departamento de Psicologia Clínica, Instituto de Psicologia, Universidade de Brasília, Campus Darcy Ribeiro, Brasília, DF 70910-900, Brazil; giardini@unb.br; 4 Programa de Engenharia Industrial, PROTEC. Escola Politécnica—PEI, Universidade Federal da Bahia, Rua Aristídes Novis, 02, 6o andar, Federação, Salvador, BA 40210630, Brazil; sartori@ufba.br; 5 Departamento de Análises Clínicas e Toxicológicas, Faculdade de Farmácia, Universidade Federal da Bahia, Salvador, Bahia 40.170-115, Brasil; rdc@ufba.br; 6 Instituto de Matemática, Universidade Federal da Bahia, Av. Adhemar de Barros, s/n, Ondina, Salvador, BA 40.170-110, Brazil; 7 Instituto de Saúde Coletiva, Universidade Federal da Bahia, Rua Basílio da Gama, s/n, Canela, Salvador, BA 40.110-040, Brazil; 8 Instituto Gonçalo Moniz (IGM), Fundação Osvaldo Cruz-FIOCRUZ-Bahia, Av. Waldemar Falcão, 121, Candeal, Salvador, BA 40.296-710, Brazil; 9 Headspace, Inc. 2415 Michigan Avenue, Santa Monica, CA 90404, USA; drmegjones@gmail.com; 10 Department of Psychiatry and Behavioral Sciences, Stanford University School of Medicine, Stanford, CA 94305, USA; btaylor@stanford.edu

**Keywords:** adolescents, school-based nutrition intervention, web-based platform

## Abstract

**Abstract:**

Interventions via the Internet are promising regarding the promotion of healthy habits among youth. The objective of this study was to evaluate the effect of an adapted version of StayingFit to promote healthy eating habits and the measurement adequacy of anthropometric markers among adolescents. A web school-based 12-month cluster-randomized controlled trial examining 7th to 9th grade students was conducted in twelve schools in Salvador, Bahia, Brazil. The schools’ students were randomly distributed into the intervention and control groups. The intervention group participated in StayingFit, an online program designed to encourage and guide healthy eating habits and control body weight. Data on food consumption, anthropometry, physical activity level, and sedentary behavior were collected from all of the students at the beginning of and after the 12-month study. Demographic and socioeconomic data were collected at baseline. The baseline data indicated high rates of overweight (14.4% overweight and 8.5% obese), insufficiently active (87.6%), and sedentary (63.7%). Furthermore, few adolescents regularly consumed fruits (18.8%) and vegetables/legumes (16.4%). Generalized estimating equations (GEEs) were used to evaluate the effect of the intervention. At the end of the follow-up period, students in the intervention group had a 43% increased chance of regularly consuming beans (OR = 1.43, 95% CIs = 1.10–1.86) and a 35% decreased chance of regularly consuming soft drinks (OR = 0.65, 95% CIs = 0.50–0.84). No differences were found between the groups studied with regard to the anthropometric parameters. Despite these modest results, the implementation of a web intervention can be beneficial and help promote positive changes in adolescent eating habits.

**Trial Registration:**

Brazilian Registry of Clinical Trials RBR-7qgnbn.

## 1. Introduction

Interventions aimed at promoting healthy behaviors using non-digital means of communication are strategies to promote health among adolescents [1,2,3,4]. Adolescence is a period of life of greater autonomy in food choices and associated with reduced physical activity [5]. Although these interventions are considered as important strategic actions, their effectiveness still presents limitations for this age group [6,7].

By contrast, adolescents who use computer-based interventions (CBIs) and, in particular, computer-tailored interventions (CTI) [8], respond better to personal motivational factors that influence behaviour [9,10,11]. In addition, these intervention modalities can be performed in combination with other actions and compose a set of comprehensive health education programs.

To align with the transformations that have occurred in contemporary society because of the increased use of computer resources, and given the potential for the application of this technology as an educational and preventive tool in the field of adolescent health, several researchers have recently been focused on the development of computerized, innovative, and specific programs for this purpose [5,12].

Different studies have been conducted to investigate the evidence regarding the efficacy of nutritional interventions to promote healthy eating habits and lifestyles using interactive media such as the Internet [13]. Changes in anthropometric parameters (e.g., weight reduction and body mass index [BMI]), increased consumption of fruits and vegetables [14,15,16], reduced fat consumption [17,18,19], and increased dairy product consumption [16] have been observed in studies of adolescents (in the intervention group) conducted by [20] and [21]. Despite these potential benefits, the results have been heterogeneous [22,23,24].

Despite the increased access to the Internet and mobile phone services, digital interventions to promote healthy eating and physical activity among this population have been used only recently in Brazil, even though the need to implement such tools for this purpose is clear [25]. Thus, introducing computerized technology to promote healthy habits and assess their effects on adolescent health and nutrition indicators (with the potential to reach large population segments at a relatively low cost) is a necessary and promising field of action [26].

Therefore, the present study evaluated the effect of an adapted version of StayingFit to promote healthy eating habits and the measurement adequacy of anthropometric markers among adolescents. The original program was developed by researchers at the School of Medicine at Stanford University, California, USA [21,27] and was made available for adaptation and the evaluation of its effectiveness in Brazil. Earlier research was effective in decreasing the BMI percentile; increasing fruit and vegetable intake; increasing the level of physical activity; while providing a reduction in the soft drinks consumption and in the adolescents’ screen time of intervention [21,27]. The indicators of satisfaction, efficiency and effectiveness evaluated the usability test and the feedback from the pre-test and the focus group demonstrated that the cultural adaptation of StayingFit may, in fact, be adequate for the population for which it is intended (publication in progress). The adapted version resulted in a CBI that is used via the Internet and uses general feedback to promote healthy eating habits and healthy weight promotion. Thus, it was hypothesized that the StayingFit Brazil would produce healthy nutrition and measurement adequacy of anthropometric markers, which would lead the intervention group to a more favorable direction when compared to the no-intervention group.

## 2. Materials and Methods 

### 2.1. Design, Population, and Sample

This 12-month community cluster trial was conducted from September 2016 to September 2017. Male and female 7th to 9th graders who were enrolled in twelve mid-sized public schools of the public comprehensive education system in Salvador, Bahia, Brazil participated in this research. The study was registered in the Brazilian Registry of Clinical Trials under the registration number RBR-7qgnbn. The methodology was designed in accordance with the recommendations of CONSORT 2010 [28].

To calculate the priori sample size, the following parameters were used: a ratio between the intervention and control participants equal to 1, a significance level of 95%, a statistical power of 0.80, and an effect size (for BMI) of 0.20 [29]. This calculation resulted in an estimate of 600 individuals. To adjust for an anticipated 20% attrition, the sample was increased to 720 students (360 in the control group and 360 in the intervention group). These calculations were performed using G*Power version 3.1.3 (Faul, Erdfelder, Lang, & Buchner, 2007).

**Figure 1:**
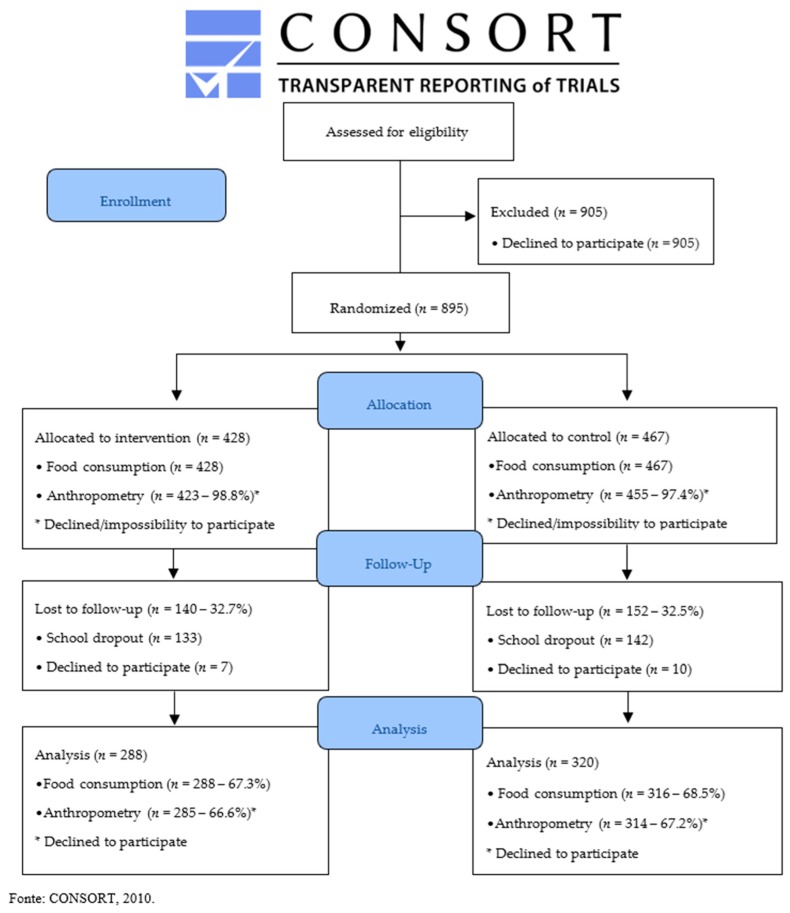
Flowchart of adolescent recruitment and participation in the study.

Twelve schools were eligible for this study (computational structure). These schools were randomly distributed into the intervention group (four schools) and the control group (eight schools). Of the 1800 students eligible for this study, 895 provided a signed informed consent document and agreed to participate in the study (Figure 1). The Ethics Committee of the School of Nutrition of the Federal University of Bahia approved this study (n 893.944/14).

### 2.2. Intervention Plan

StayingFit is an online program organized to encourage and guide weight control and healthy eating habits [21]. It is based on the tenants of Cognitive-Behavioral Therapy (CBT) [21,30]. It integrates techniques and concepts from two main approaches: the cognitive therapy, with techniques destined to the correction of beliefs and dysfunctional thoughts; and the behavioral therapy, used to modify inappropriate behaviors. StayingFit Brazil adopted a generalist form of feedback and used messages guided by the Food Guide for the Brazilian Population [31] and the recommendations of the World Health Organization (WHO) concerning aspects related to the practice of physical activity [32]. After completing food frequency and physical activity questionnaires, automated feedback was immediately displayed on the computer screen. StayingFit Brazil was made available to the participants in the control group after it was implemented in the intervention schools.

The program also includes the participation of parents and teachers. Parents received printed material with the content of the program sessions. Teachers also got involved with the “StayingFit Brazil FAQs” tool, which is available on the “StayingFit Brazil” site (http://stayingfitbrasil.ufba.br/) and contains information on the program’s content.

Because StayingFit is an American program, Barrera et al. (2013) and Castro et al. (2015) culturally adapted it to the cultural standards, meanings, and values of Brazil via a systematized process that considered language, culture, and context [33,34]. The adapted version was made available in the computer labs of each school in the intervention group, and a nutritionist and assistant (i.e., nutrition student) supervised the implementation of the program.

### 2.3. StayingFit Brazil Intervention Components

This intervention program contains 16 independent sessions. The prerequisite for performing the subsequent session was to complete the previous session. Participants were encouraged to read through the content, complete the exercises, logs, and feedback from the sessions. The components of StayingFit Brazil are summarized in Table 1. The topics/content of StayingFit Brazil are presented in Table 2.

StayingFit Brazil was delivered through a virtual learning environment (AVA) [35]. To create this AVA, Moodle (https://moodle.org/) was chosen because it is free, multilingual, flexible, and customizable software and because it is the official learning platform of the Federal University of Bahia (UFBA).

Thus, each participant was given a username and password to log in and participate in the program. The research team defined each user’s name and password based on their unique identification numbers to ensure the individuality and confidentiality of participants’ information. The students were unaware of their usernames and passwords to prevent the use of the program without the supervision of the project team and ensure the privacy of their information. The activities of StayingFit Brazil were developed in full-time schools, in the afternoon shift in order not to harm the progress of compulsory activities. Students accessed the sessions once a week for 30 min in the computer room of each of the schools in the intervention group and was supervised by a nutritionist and an assistant (nutrition student) to help adolescents to use the program and overcome technical difficulties of access.

### 2.4. Data Collection

All students across the twelve schools received anthropometric measurements, and information concerning sexual maturity, food consumption, physical activity, and level of sedentarism were collected at the beginning of the study and after 12 months. Information about the socioeconomic conditions of the students’ families was obtained at baseline.

#### 2.4.1. Food Consumption

Food consumption over the last 7 days was measured based on the self-reported consumption frequency of food using the following food groups or preparation categories: beans, at least one type of vegetables, raw salad, cooked vegetables or legumes, fruits, milk, soft drinks, candies, cookies, crackers, snacks, fried snacks, and ultraprocessed meats [36]. The first six items were considered as healthy and adequate food markers, whereas the last seven were considered as markers of unhealthy diets [37,38]. The consumption of these foods was expressed regular consumption (adolescents who consumed them at least 5 of the 7 days before the study) or less frequent consumption (adolescents who consumed them between 0 and 4 days of the 7 days prior to the study) [37].

#### 2.4.2. Anthropometric Data

All measurements followed the procedures recommended by the Anthropometric Standardization Reference Manual [39]. Body weight was obtained using a Marte^®^ portable scale (Marte Balanças e Aparelhos de Precisão, São Paulo, Brazil). Height was measured using a Seca Leicester Height Measure^®^ stadiometer (Seca, Hamburg, Germany). BMI or the Quetelet Index (P/E2) was calculated using the relationship between weight (kg) and height squared (m^2^). The cut-off points were expressed as percentiles for BMI, according to sex and age, which is recommended by the WHO (2007) [40] to classify the nutritional status of adolescents. Individuals with BMI-for-age lower than the 3rd percentile were classified as malnourished; those with a value greater than or equal to the 3rd percentile but below the 85th percentile were classified as eutrophic; and those with a value equal to or greater than the 85th percentile were classified as overweight (where greater than 85th percentile but less than 97th percentile was classified as overweight, and greater than or equal to 97th percentile was classified as obese). Waist circumference (WC) was measured using a Seca^®^ fibreglass inelastic tape (Seca, Hamburg, Germany). with a centimeter scale applied at the midpoint between the last rib and the upper iliac crest of the adolescent. The waist-height ratio (WHR) was obtained by dividing WC (cm) by height (cm) [41]. Hip circumference (HC) was measured at the level of the maximum posterior extension of the buttocks. Arm circumference (AC) and tricipital skinfold (TSF) thickness were measured at the midpoint between the acromion and the olecranon of the left or non-dominant arm. Subscapular skinfold (SSSF) thickness was measured at the inferior angle of the scapula. A Lange^®^ scientific body fat caliper with a precision of 0.1 mm was used to obtain TSF and SSSF (Lange Skinfold Caliper, Cambridge Scientific Industries, Inc., Cambridge, MD, USA). 

The researchers were instructed to perform a third measurement when the first two measurements were inconsistent. Nevertheless, two measurements were recorded for each variable, and the mean between the measurements was taken as the final value.

#### 2.4.3. Level of Physical Activity and Sedentarism

The overall physical activity indicator was used to assess the physical activity level of adolescents [36]. Those who accumulated 300 mins or more of weekly physical activity were considered as active, whereas those who accumulated between 1 and 299 min of weekly physical activity were considered as insufficiently active [32]. Sedentary habit was evaluated based on the time spent in sedentary activities, which was considered as the number of hours per day in which the adolescents watching television, using the computer, playing videogames and doing other activities. In this case, the question was, “On an ordinary weekday, how long do you spend sitting, watching TV, using the computer, playing videogames, talking with friends, or doing other activities? (not including Saturdays, Sundays, holidays, and time sitting at school)”. Participants who reported habitually spending 2 or more hours of screen time per day were considered as showing sedentary behavior [42]. Although not validated, the questionnaires were widely used in the studies of the National Adolescent School-Based Health Survey (PeNSE). The PeNSE is a school-based national epidemiological survey, conducted by the Brazilian Institute of Geography and Statistics (IBGE) in collaboration with the Ministry of Health (MS) to monitor the health conditions of Brazilian adolescents [36].

#### 2.4.4. Sexual Maturity Data

The onset of pubescence among females is classified based on stage II breast development, and the end (post-pubescence) of the growth spurt is characterized based on age at menarche. For males, the onset of the growth spurt (pubescence) is indicated as Tanner stage III, and final (post-pubescence) development is indicated by stage IV genitalia development [43].

The identification of these stages was made by the students’ self-description with the aid of portraits provided by the interviewers. Schematics that reproduced the different stages of puberty were used to self-evaluate the characteristics of the breasts, the onset age of menarche, and pubic hair growth among girls; schematics were also used to evaluate genitalia and pubic hair growth among boys [44,45]. As such, students were classified into three categories: pre-pubertal, pubertal, and post-pubertal.

#### 2.4.5. Demographic and Socioeconomic Data

In addition to gender and age, socioeconomic data such as mother’s and/or head of household’s education (Illiterate or incomplete primary education/completed primary education or incomplete secondary education/completed secondary education or incomplete higher education/completed higher education) and assets were collected. A household assets score was constructed based on having a bathroom, a salaried maid, an automobile, a microcomputer, a dishwasher, a refrigerator, a freezer, a washing machine, a DVD player, a microwave, a motorcycle, and a dryer according to the questionnaire of the Brazilian Association of Research Companies-Associação Brasileira de Empresas de Pesquisa: ABEP (2013). Although it is not a validated questionnaire, it is widely used in Brazilian studies to evaluate the interviewees’ economic status. Each item received a weight equivalent to the inverse of the frequency of its possession or presence in the sample studied. A score for each adolescent was obtained by adding the weights of the respective items. The distribution of the score was categorized into tertiles with respect to the distribution observed in the sample according to a previously described method [37]. 

#### 2.4.6. Data Entry

EpiData version 3.0 (EpiData Association, Odense, Denmark) was used to process and construct the database. After reviewing the questionnaires and correcting errors that resulted from the coding initially performed in the field, the data were re-entered. The simple frequencies of the variables were verified, and the consistency between questions and answers were examined via instruments used to clean the data.

#### 2.4.7. Statistical Analyses

Descriptive analyses were conducted to characterize the sample. Proportions were used to describe the categorical data, and means (standard deviations) were used to describe the continuous variables. Chi-square and Student’s t-tests were used to identify the possible differences between the intervention and control groups at baseline. The response variables were the changes in the anthropometric indicators and the frequency of food consumption. In addition, the main independent variable was use of the intervention (yes/no). Generalized estimating equations (GEEs) were used to evaluate the influence of the intervention program on the changes in anthropometric profile and food intake. GEE is a technique appropriately used for repeated measures because it reflects the relationship between a continuous or binary response variable and its corresponding predictor variables; moreover, it considers the correlation between the measures at each moment in time [46]. The regression parameters (β and OR) were estimated using the pseudo-likelihood method with the robust option to correct the standard error in situations involving repeated observations.

Possible confounds were explored using the theoretical framework available in the literature [1,47,48] and the dataset of this study. The analyses adjusted for the following variables: sex, age, economic condition, caregiver education, physical activity level, and pubertal development [2,15,49]. Two-tailed tests and a significance level of 5% were used. The analyses were performed using Stata 14.0 (Stata Corporation, College Station, TX, USA).

## 3. Results 

The initial sample (baseline) was composed of 895 students (428 in the intervention group; 467 in the control group; Figure 1), which is a larger number than the sample size calculated, but these participants were maintained in the analysis for ethical reasons. Regarding the demographic and pubertal development characteristics of the sample, the mean age of the adolescents was 14.5 years (1.42), 51.6% were male, and the majority were in the post-pubertal stage (85.7%). In addition, 27.0% of the adolescents belonged to families whose guardians had low education levels (illiterate/incomplete elementary education). Regarding nutritional status, 5.9% of the participants were malnourished, 71.2% were eutrophic, and 22.9% were overweight (14.4% overweight and 8.5% obese). Regarding the levels of physical activity and sedentarism, 87.6% were considered as insufficiently active, and 63.7% were sedentary (68.8%, intervention and 59.0%, control; *p* = 0.002). Additional information is shown in Table 3. The two groups significantly differed with regard to the following anthropometric characteristics and food consumption variables at baseline: weight (*p* = 0,043), WC (0,015), AC (*p* = 0.002), and soft drink consumption (*p* = 0.009; Table 4).

To evaluate the effect of the intervention, 285 (66.6%) and 314 (67.2%) of the participants in the intervention and control groups, respectively, had anthropometric parameters, and 288 (67.3%) and 316 (68.5%) of the participants in the intervention and control groups, respectively, provided food consumption data (see Figure 1). The characteristics of the participants were similar to those of the dropouts with the exception of age (14.25 (1.22) years vs. 14.99 (1.66) years, respectively; *p* < 0.001), weight (54.48 (13.61) kg vs. 56.44 (13.15) kg, respectively; *p* = 0.044), AC (24.47 (4.02) cm vs. 25.08 (3.85) cm, respectively; *p* = 0.033), and regular consumption of milk (38.18% vs. 30.697%, respectively; *p* = 0.029; Supplementary Tables S1 and S2). Losses due to follow-up were expected and occurred primarily because of school dropout and refusal to participate in the second stage of the study.

There was an increase in the anthropometric parameters in both groups studied throughout the follow-up, except for arm circumference and waist-to-height ratio in the intervention group; Moreover, there was no difference at the end of the study between both groups. In both groups, there was a reduction in the regular consumption of soft drink, but with greater expressiveness in the intervention group. In the intervention group, there was a reduction in the regular consumption of crackers, cookies, candies and milk (*p* < 0.05). Besides that, there was a difference at the end of the study between both groups for regular consumption of cooked vegetables or legumes, milk, crackers and soft drinks (Table 4). However, some differences were not maintained in more robust analyzes.

Generalized estimating equations (GEEs) were used to explore the effect of the intervention. At the end of the follow-up, the chance of consuming beans increased by 43% (OR = 1.43, 95% CIs = 1.10–1.86), and the chance of regularly consuming soft drinks reduced by 35% (OR = 0.65, 95% CIs = 0.50–0.84) among adolescents who underwent the intervention. No significant differences were found with regard to the anthropometric parameters studied. All analyses were adjusted for age, gender, level of physical activity, household assets score, caregiver education and pubertal development (Table 5 and Table 6).

## 4. Discussion

The goal of this study was to evaluate an adapted version of StayingFit, a CBI promoting healthy eating and weight control among adolescents. The baseline data indicated high rates of overweight individuals (14.4% overweight and 8.5% obese), insufficiently active (87.6%), and sedentary lifestyles (63.7%) Furthermore, fewer adolescents regularly consumed fruits (18.8%) and vegetables/legumes (16.4%). This scenario, similar to that of the Brazilian youth and adolescents from other countries in the last decades [50,51,52], indicates an urgent need to motivate adolescents to adopt healthy eating and lifestyle habits, such as physical activity, as a strategy for reducing risk factors and chronic non-communicable diseases already at this stage of life [5,6,7]. 

Some limitations of non-digital intervention programs, such as low motivation of the participants, lack of time for the intervention, high costs, among others, can be minimized in interventions that use digital technology, placing them as an alternative to face-to-face interventions [21]. Hence, we hypothesized that the StayingFit Brazil leads to positive outcomes in healthy nutrition and measurement adequacy of anthropometric markers. In line with the hypothesis, positive effects found after 1 year of intervention appears to be modest. 

Generalized estimating equations (GEEs) were used to explore the effect of the StayingFit Brazil. Compared with the control, the intervention was associated with reduced consumption of soft drinks (OR = 0.65; 95% CI 0.50–0.84) and greater consumption of beans (OR = 1.43; 95% CI 1.10–1.86). These results suggest that eating habits characteristic of this age group may be modified with this type of intervention. In turn, no differences in the observed anthropometric parameters were observed, after adjustment of the estimates by confounding factors. These results are consistent other universal prevention programs interventions which have also failed to find changes in BMI and other adiposity measurements in adolescents [15,16].

Although our intervention did not seem to have strong enough effects on the target behaviors to affect anthropometric parameters, the increased odds of regular consumption of beans (43% increase) associated with the intervention is noteworthy. This is a leguminous traditionally found in the diet of the Brazilian population, and its consumption is strongly associated with protection against several diseases [53,54]. This leguminous is an important source of fibre, protein, folate, zinc, and other nutrients, and beans combined with rice is a meal with adequate nutritional value. Studies point to a reduction in the frequency of beans consumption among adolescents over the last 10 years. For instance, results from the National Adolescent School-based Health Survey (PeNSE) performed in 2009 and 2015 show a reduction by a little over 10% in the proportion of students that regularly consume beans [36,55]. Adopting strategies that encourage beans consumption is necessary on account of the benefits presented by this leguminous. The intervention was also associated with lower odds of regular consumption of soft drinks (reduction of 35%). This is an important result in light of the effect of sugary drinks on obesity [56,57]. The consumption of sugary drinks (including soft drinks) has been considered an obesity-promoting factor and reducing their consumption has been identified as an important measure for controlling weight gain and, consequently, the development of chronic non-communicable diseases in children and adolescents [58]. 

Even if studies in this field have similar goals (i.e., evaluating the effects of computerized interventions to promote healthy eating and anthropometric parameters among adolescents), differences exist regarding the intervention methodologies used (e.g., the study design, intervention type (CBI or CTI)), duration of intervention and follow-up, number and/or frequency of educational sessions held), which compromises the comparison of the results. 

Although they are modest, our findings corroborate the positive effect of similar web-based strategies on reducing the consumption of sugary drinks [15]. Ezendam and colleagues (2012) showed that the FATaintPHAT intervention conducted with Dutch adolescents was associated with a lower likelihood (OR = 0.54, 95% CIs = 0.34 to 0.88) of consuming more than 400 mL of sugary drinks per day during the first 4 months of follow-up. They also showed a higher consumption of vegetables (in grams/day; β = 19.34, 95% CIs = 7.54 to 31.21) compared with their control [15]. The meta-analysis conducted by Vargas Garcia et al. (2017) showed that participants who receive intervention (via randomized controlled trials (RCTs), cluster RCTs, and non-RCTs) consume 66 mL less sugary (sugar-sweetened or diet) soft drinks (95% CIs = −130 to −2.0 *p* = 0.04) [59]. Previous intervention studies have not evaluated the consumption of legumes via the web, which highlights the novelty of the current study. 

By contrast, previous interventions have shown the positive effects of web-based on fruit, vegetable, and fat consumption [14,16,18,20,60] as well as anthropometric markers [20]. Mauriello et al. (2010) evaluated of the Health in Motion CTI performed among American adolescents and observed a greater consumption of fruit and vegetables 2 months (3.86 vs. 3.0), 6 months (3.55 vs. 2.73), and 12 months (3.67 vs. 2.97) after the intervention (*p* < 0.001) [14]. Chamberland et al. (2017) [16] demonstrated a higher consumption of daily servings of fruits and vegetables in the intervention group via the Team Nutriathlon CTI for Canadian adolescents compared with a control group (*p* ≤ 0.05). Haerens et al. (2006) developed a CTI using a self-explanatory CD-ROM and indicated a significantly higher reduction in fat intake in the intervention group (−20 g/day) compared with the control group (−10 g/day, *p* < 0.05). Likewise, the percentage of energy from fat was significantly reduced by 9% in the intervention group and by 5% in the control group (*p* < 0.001); however, this favorable outcome was observed for girls only [17]. The same results were reiterated in later publications [18,19]. Haerens et al. (2006) found significantly lower increases in BMI (F = 12.52, *p* < 0.05) and the BMI z-score (F = 8.61, *p* < 0.05) in the intervention group, and a positive result was found only among girls [20]. Because the few existing studies focus only on the transfer of nutritional knowledge via the web, a strong need exists for studies to investigate whether computer-based nutrition education tools are beneficial in supporting students’ abilities to translate their acquired nutritional knowledge into nutritional behavior.

A possible explanation for the modest findings of this study includes the adopted education format (computer-based intervention (CBI)), which is a modality little explored in nutrition education [10]. There is a consensus among health communication scholars that personalized information is more effective for motivating the adoption of health protection behaviors than is general communication, which uses undifferentiated messages. However, all communication provided by StayingFit was based on messages focusing on the promotion of a healthy lifestyle in adolescents (rather than treatment mode). 

Another explanation may be related to the number of evaluated outcomes. Results of a systematic review to evaluate the effect of digital nutritional interventions failed when focused on multiple outcomes [23]. The complexity of the intervention in question should also be considered. Corroborating this hypothesis, a review on the prevention of risk factors for cardiovascular disease concluded that programs addressing multiple health behaviors were not effective at reducing obesity among children [61]. 

In addition, the participation of parents was modest: a systematic review of digital interventions for the promotion of healthy eating and physical activity among adolescents showed that the interventions produced a greater effect when they included the active participation of parents [24]. Considering the changes assessment was done based on the application of self-filled questionnaires pre- and post-intervention, instead of direct observation of such behaviors, one cannot discard the possibility that in the post-intervention, the students could have answered according to what they learned during the activities developed in the program and not according to what they actually did. It is important to highlight the possibility that the control group’s school had developed its own initiatives to promote health; for ethical reasons, no attempt was made to prevent it.

### Strengths and Limitations of the Study

This study has as merit in its novelty: It is the first to evaluate the effect of a CBI to promote healthy habits and nutrition indicators among adolescents in Brazil. The following methodological aspects of this study ensure that the results are robust: (i) the randomized clinical trial design reduced the interference of possible confounds; (ii) StayingFit was designed to encourage adolescents to use the technology in a smart and effective manner, promoting healthy behaviors and lifestyles in a way that they would not be harmed by increasing the time that they spend in front of the screen; (iii) instead, the program was implemented during school hours and did not contribute to an increase in sedentary behavior; (iv) In addition, the StayingFit had a high participation rate, 85% of the adolescents completed the 16 sessions of the program. 

In turn, some limitations of the study should be considered. Despite the efforts to locate the children in the school, there was a significant number of losses during follow-up—approximately 30%. Results were similar to those of Mauriello et al. (2010) and Maes et al. (2011), with 27% and 48% of losses during the follow-up, respectively [14,57]. However, they were higher than the results found in the original StayingFit, with 15% of losses during follow-up [20]. Such losses were mainly due to school dropout and refusal to participate in the second phase of the project. According to the school census, the percentage of school dropouts in Salvador in 2014 and 2015 was 5.9%, and this percentage was 6.1% for students from public schools [62]. Among the factors that contribute to this problem are distance from school in association with lack of school transportation; precarious socioeconomic situation, which leads students to start working before they even finish school; violence in schools; lack of interest; diseases/learning difficulties of the students; and lack of school structure [63]. In turn, the study design, the balance of losses between the intervention and control groups, and the homogeneity of the study population contribute to the mitigation of eventual biases. Considering that the assessment of changes in habits was based on self-report questionnaires completed during the pre- and post-intervention periods instead of direct observation of these behaviors, one cannot ignore the possibility that the students provided answers that were consistent with what they had learned in the program activities but not with what they were actually doing. 

## 5. Conclusions

The results of the present study suggest a positive effect of the adapted version of StayingFit with regard to an increase in the frequency of beans consumption and a decrease in the frequency of soft drink consumption among elementary school students. It appears that implementing a web-based intervention would be beneficial and could be used to promote changes in eating habits and health among adolescents. Even though StayingFit is not a multi-level intervention, it can be integrated into a school environment that has implemented a nutritional education program in the curriculum or increased the healthy food availability in its environment. Certainly, the challenge is to assure the maintenance of these results over time. Thus, the development of strategies to promote the maintenance of these results is clearly a relevant aspect of the research agenda of this field of knowledge. It has been shown that chronic non-communicable diseases represent the greatest burden of disease in the country, and actions to promote health in the child and adolescent are of the utmost importance so that healthy habits in childhood and adolescence are maintained throughout life.

## Figures and Tables

**Table 1:** Components that structure the virtual learning environment of *StayingFit* Brazil.

Components	Description
Thematic Sessions	Each session contains 10 to 15 pages of online content with texts aimed at 7th and 8th grade reading level and that can be read in approximately 30 min.
Learning questions	At the end of each session, students answered questions about their learning on that week’s topic. The questions (multiple choice and open-ended answers) evaluated the assimilated knowledge.
Session feedback	These questions assess knowledge, attitudes, behaviors, and self-efficacy related to the specific skills taught during the session. To assess student involvement and achievement, students evaluated the levels at which the content was useful, interesting, and fun.
Food Log	Program users completed an online food-intake questionnaire regarding the last 7 days. After the adolescents had submitted their food frequency data, the participants received automated (general) feedback aligned with the recommendations of the Food Guide for the Brazilian Population, 2014 [31].
Physical activity log	The adolescents reported how often they had physical activity over the last 7 days. They received automated (general) feedback on the adequacy of their physical activity aligned with the recommendations of the WHO [32].
Hunger and satiety scale	A hunger/satiety scale ranging from 0 (hungry/voracious) to 10 (satiety) was used to teach adolescents to attend more to their appetite. Participants were encouraged to monitor their levels of hunger throughout the day, to start eating when their internal signs of appetite reached a hunger level of 3, and to stop eating when they reached a level 7.
Goals log	Adolescents were encouraged to record their goals regarding food consumption and physical activity.
Discussion forum	The adolescents were invited to anonymously discuss issues related to the program material in a discussion forum.
Material for parents	Parents received printed material of the content of the sessions given to students.
Material for teachers	“*StayingFit* FAQs” (available on the *StayingFit* Brazil website) was made available to teachers, who were encouraged to contact the research team if they had questions.

**Table 2:** Topics/content of the *StayingFit* Brazil sessions.

1	Introduction to the program: Internet etiquette; reasons to adopt healthy eating habits and lifestyles
2	Introduction to healthy eating practices; classifying food groups (red, yellow, and green); defining serving sizes and "flexible eating"
3	Moving the body: importance, strategies to increase physical activity, daily recommendations, and variety
4	Healthy lifestyle: routines beneficial to the body and how to overcome barriers to healthy eating
5	Binge eating: definition and triggers; how to identify and monitor the signs of hunger and satiety; distinction between healthy and unhealthy snacks; definition of goals for healthy eating
6	Weight stigma: Why weight stigmas are harmful and how to stay confident in the face of this problem
7	Labels: understanding the components of food nutritional labels; healthy eating away from home; the harmful effects of sugary drinks
8	Eating disorders; reflecting on food myths; warning signs for risky behaviors; ways to stay healthy
9	Signs of the body; identifying good practices that lead to eating more slowly; identifying practices of conscious eating; reflecting on emotional eating
10	Body image components that comprise self-esteem; the direct and indirect triggers of negative thoughts and feelings that affect body image
11	Barriers to the adoption of healthy eating; planning a healthy lunch box
12	Overcoming difficulties; barriers to exercise
13	Food planning: Why do diets not work? The negative consequences of diets; eating disorders
14	“Fad diets”: The influence of the media. The importance of making healthy, smart, and informed decisions; the benefits of water consumption
15	Review of the *StayingFit* content
16	Final review; encouraging the maintenance of healthy habits over the long term

**Table 3:** Characterization of the study groups at baseline. *Salvador*-BA, 2016.

Variables	Baseline		^g^*p* Value	Total (%)
	Intervention *n* = 428	Control *n* = 467		
**Age (years) ^a^**—mean (SD)	14.48 (1.43)	14.50 (1.42)	0.847	14.49 (1.42)
**Gender (%) ^a^**			0.178	
Male	54.0	49.5		51.6
Female	46.0	50.5		48.4
**Pubertal development (%) ^b^**			0.703	
Pre-pubertal	6.9	6.9		6.9
Pubertal	8.1	6.6		7.4
Post-pubertal	84.9	86.5		85.7
**Household assets score (%) ^c^**			0.479	
Low	33.2	35.8		34.6
Medium	34.4	30.6		32.4
High	32.4	33.6		33.0
**Caregiver Education (%) ^d^**			0.317	
Illiterate/Incomplete primary education	24.0	29.7		27.0
Complete primary education/Incomplete secondary education	25.5	23.1		24.2
Complete secondary education/Incomplete higher education	44.2	41.3		42.6
Complete higher education	6.3	5.9		6.1
**Anthropometric status (%) ^e^**			0.430	
Malnutrition	5.9	5.9		5.9
Normal weight	69.9	72.4		71.2
Overweight	14.1	14.7		14.4
Obesity	10.1	7.0		8.5
**Physical activity (%) ^f^**				
Insufficiently active	89.2	86.3	0.183	87.6
Physically active	10.8	13.7		12.4
**Sedentary behavior (%) ^f^**			**0.002**	
Sedentary (screen-time ≥ 2 h)	68.8	59.0		63.7
Not Sedentary (screen-time < 2 h)	31.2	41.0		36.3

^a^ Gender, age *n* = 895; ^b^ Pubertal development *n* = 881; ^c^ Household assets score *n* = 853; ^d^ Caregiver Education *n* = 855; ^e^ Anthropometric status *n* = 882; ^f^ Physical activity and sedentary behavior *n* = 892. ^g^ Age by student test and categorical data by Chi-square test.

**Table 4:** Outcome Measures for the Intervention and Control Groups in baseline and follow-up. Salvador, 2017.

Outcome	Baseline	Follow-up	Difference at Follow-up and Baseline ^a^	Difference at the End of the Study ^b^
**Anthropometrics (mean (SD))**				
**Weight (kg)**				0.070
Intervention	56.05 (14.22) (*n* = 425)	58.91 (14.15) (*n* = 286)	**2.86**	
Control	54.22 (12.71) (*n* = 459)	56.90 (13.02) (*n* = 317)	**2.68**	
**BMI (kg/m^2^)**				0.293
Intervention	20.56 (4.35) (*n* = 425)	20.95 (4.17) (*n* = 285)	**0.39**	
Control	20.22 (3.89) (*n* = 457)	20.61 (3.79) (*n* = 317)	**0.39**	
Waist circumference (cm) *				0.393
Intervention	70.34 (10.28) (*n* = 425)	71.45 (10.38) (*n* = 286)	**1.11**	
Control	68.78 (8.75) (*n* = 456)	70.78 (8.73) (*n* = 314)	**2.00**	
Hip circumference (cm) *				0.661
Intervention	88.87 (10.3) (*n* = 423)	89.16 (9.40) (*n* = 286)	**0.29**	
Control	88.27 (9.17) (*n* = 455)	89.50 (9.57) (*n* = 314)	**1.23**	
Arm circumference (cm) *				0.137
Intervention	25.09 (4.11) (*n* = 425)	24.99 (3.97) (*n* = 285)	−0.10	
Control	24.27 (3.80) (*n* = 455)	25.46 (3.82) (*n* = 315)	**1.19**	
**Triceps skinfold (mm)**				0.499
Intervention	14.07 (6.86) (*n* = 423)	14.82 (7.70) (*n* = 286)	**0.75**	
Control	13.76 (6.48) (*n* = 455)	14.41 (7.10) (*n* = 315)	**0.65**	
**Subscapular skinfold (mm)**				0.547
Intervention	14.71 (7.20) (*n* = 423)	15.89 (7.33) (*n* = 286)	**1.18**	
Control	14.50 (6.74) (*n* = 455)	15.53 (7.27) (*n* = 314)	**1.03**	
**Sum of two folds (mm)**				0.505
Intervention	28.68 (13.40) (*n* = 423)	30.68 (14.32) (*n* = 286)	**2.00**	
Control	28.28 (12.67) (*n* = 455)	29.91 (13.69) (*n* = 314)	**1.63**	
**Waist-to-height ratio**				0.993
Intervention	0.43 (0.06) (*n* = 425)	0.43 (0.57) (*n* = 285)	0.00	
Control	0.42 (0.05) (*n* = 456)	0.43 (0.48) (*n* = 314)	**0.01**	
**Diet** (% regular consumption > 5 days of the 7 days prior to the study)				
**Beans**				0.077
Intervention	25.47 (*n* = 428)	25.44 (*n* = 287)	−0.03	
Control	30.62 (*n* = 467)	31.96 (*n* = 316)	1.34	
**At least one type of vegetables**				0.104
Intervention	15.19 (*n* = 428)	16.72 (*n* = 287)	1.53	
Control	17.56 (*n* = 467)	21.97 (*n* = 314)	4.41	
**Raw salad**				0.619
Intervention	16.12 (*n* = 428)	15.38 (*n* = 286)	−0.74	
Control	16.49 (*n* = 467)	16.88 (*n* = 314)	0.39	
**Cooked vegetables or legumes**				**0.050**
Intervention	10.75 (*n* = 428)	10.10 (*n* = 287)	−0.65	
Control	9.85 (*n* = 467)	15.46 (*n* = 317)	5.61	
**Fruits**				0.367
Intervention	19.16 (*n* = 428)	17.13 (*n* = 286)	−2.03	
Control	18.63 (*n* = 467)	20.00 (*n* = 315)	1.37	
**Milk**				**0.038**
Intervention	37.62 (*n* = 428)	31.94 (*n* = 288)	**−5.68**	
Control	34.05 (*n* = 467)	40.06 (*n* = 317)	6.01	
**Fried Snacks**				0.835
Intervention	10.75 (*n* = 428)	10.42 (*n* = 288)	−0.33	
Control	10.92 (*n* = 467)	9.90 (*n* = 313)	−1.02	
**Ultraprocessed meats**				0.809
Intervention	11.92 (*n* = 428)	10.18 (*n* = 285)	−1.74	
Control	10.06 (*n* = 467)	9.58 (*n* = 313)	−0.48	
**Crackers**				**0.022**
Intervention	30.61 (*n* = 428)	18.06 (*n* = 288)	**−12.55**	
Control	25.27 (*n* = 467)	25.80 (*n* = 314)	0.53	
**Cookies**				0.242
Intervention	27.80 (*n* = 428)	21.95 (*n* = 287)	**−5.85**	
Control	24.63 (*n* = 467)	26.03 (*n* = 315)	1.40	
**Snacks**				0.839
Intervention	13.79 (*n* = 428)	9.79 (*n* = 286)	−4.00	
Control	10.71 (*n* = 467)	10.29 (*n* = 311)	−0.42	
**Candies**				0.305
Intervention	36.21 (*n* = 428)	25.69 (*n* = 288)	**−10.52**	
Control	34.90 (*n* = 467)	29.43 (*n* = 316)	−5.47	
Soft drinks *				**0.041**
Intervention	33.41 (*n* = 428)	25.09 (*n* = 287)	**−8.32**	
Control	25.48 (*n* = 467)	18.21 (*n* = 313)	**−7.27**	

^a^ Anthropometrics measures by paired *t*-test and diet by McNemar test; ^b^ Anthropometrics measures by student test and diet by Chi-square test; * Significantly difference of the study groups at baseline (*p* < 0.05).

**Table 5:** Estimates (β) and standard errors to assess the effects of a program *StayingFit* on the anthropometric parameters of study adolescents. *Salvador*-BA, 2017.

Models	Estimate #	Standard Error	*p* #
**Weight (Kg)**			
Intercept	16.24	1.08	0.000
Intervention	0.97	0.75	0.201
**BMI (kg/m^2^)**			
Intercept	14.31	1.08	0.000
Intervention	0.26	0.25	0.293
**WC mean (cm)**			
Intercept	51.95	2.63	0.000
Intervention	1.00	0.58	0.087
**HC mean (cm)**			
Intercept	71.34	2.67	0.000
Intervention	0.35	0.62	0.566
**AC mean (cm)**			
Intercept	17.51	1.28	0.000
Intervention	0.30	0.25	0.229
**TSF mean (mm)**			
Intercept	7.17	1.82	0.000
Intervention	0.54	0.40	0.180
**SSSF mean (mm)**			
Intercept	3.54	1.34	0.068
Intervention	0.38	0.42	0.369
**S2SF mean (mm)**			
Intercept	9.62	3.40	0.005
Intervention	0.79	0.78	0.306
**WHR**			
Intercept	0.40	0.804	0.000
Intervention	0.00	0.033	0.185

# Generalized estimating equations (GEEs) adjusted by gender, age, household assets score, caregiver education, time of physical activity and pubertal development. BMI: Body mass index; WC: Waist circumference; HC: Hip circumference; AC: Arm circumference; TSF: tricipital skinfold; SSSF: Subscapular skinfold; S2SF: Sum of 2 folds; WHR: The waist-height ratio.

**Table 6:** Odds ratios (ORs) and corresponding confidence intervals (95% CIs) to assess the effect of a program *StayingFit* on food consumption among study adolescents. Salvador-BA, 2017.

Groups	Food Groups							
	Model 1 ^&^		Model 2 ^&^		Model 3 ^&^		Model 4 ^&^	
	Beans		At least one type of vegetables		Raw Salad		Cooked vegetables or legumes	
	OR	95%CI	OR	95%CI	OR	95%CI	OR	95%CI
Intervention	**1.43**	**1.10–1.86**	1.26	0.94–1.69	1.08	0.79–1.48	1.10	0.77–1.57
Control	1	-	1	-	1	-	1	-
	Model 5 ^&^		Model 6 ^&^		Model 7 ^&^		Model 8 ^&^	
	Fruits		Milk		Fried Snacks		Ultraprocessed meats	
	OR	95%CI	OR	95%CI	OR	95%CI	OR	95%CI
Intervention	1.00	0.75–1.34	1.06	0.83–1.36	0.98	0.68–1.42	0.83	0.58–1.20
Control	1	-	1	-	1	-	1	-
	Model 9 ^&^		Model 10 ^&^		Model 11 ^&^		Model 12 ^&^	
	Cracker		Cookies		Snacks		Candies	
	OR	95%CI	OR	95%CI	OR	95%CI	OR	95%CI
Intervention	1.00	0.78–1.30	0.97	0.74–1.26	0.80	0.56–1.16	1.01	0.78–1.30
Control	1	-	1	-	1	-	1	-
	Model 13 ^&^ Soft drinks							
	OR	95%IC						
Intervention	**0.65**	**0.50–0.84**						
Control	1	-						

^&^ Generalized estimating equations (GEEs). Models adjusted by gender, age, household assets score, caregiver education, time of physical activity and pubertal development.

## Supplementary Materials

The following are available online at https://www.mdpi.com/1660-4601/16/10/1674/s1, Table S1: Loss analyses between participants and dropouts of the study. Salvador, 2017, Table S2: Loss analyses between participants and dropouts of the study considering the intervention and control group. Salvador, 2017.
