# Supplementary material for: Evaluation of the Computer-Based Intervention Program Stayingfit Brazil to Promote Healthy Eating Habits: The Results from a School Cluster-Randomized Controlled Trial
Source: Int J Environ Res Public Health. 2019 May 14;16(10):1674. doi: 10.3390/ijerph16101674 (PMC6572183; doi:10.3390/ijerph16101674)
Supplement: Supplementary file 1 [file ijerph-16-01674-s001.pdf]

Supplementary Materials:

Table S1. Loss analyses between participants and dropouts of the study. Salvador, 2017.

Variables	Participants		Dropouts		p
	% / μ (SD)	N	% / μ (SD)	N	
**Age (in years)**[a] - mean (SD)	14.25 (1.22)	605	14.99 (1.66)	290	**0.000**
**Weight (kg)**[b] - mean (SD)	54.48 (13.61)	601	56.44 (13.15)	283	**0.044**
**Waist circumference (cm)** [c]- mean (SD)	69.30 (9.94)	600	70.03 (8.64)	281	0.287
**Hip circumference (cm)** [d] - mean (SD)	88.17 (9.83)	600	89.41 (9.48)	278	0.078
**Arm circumference (cm)** [e] - mean (SD)	24.47 (4.02)	600	25.08 (3.85)	280	**0.033**
**Triceps skinfold (mm)** [d] - mean (SD)	13.86 (6.73)	597	14.01 (6.54)	281	0.753
**Subscapular skinfold (mm)** [d] - mean (SD)	14.55 (7.09)	597	14.70 (6.69)	281	0.767
**Sum of two folds (mm)** [d] - mean (SD)	28.37 (13.21)	597	28.70 (12.60)	281	0.725
**Waist-to-height ratio** [c] - mean (SD)	0.42 (0.05)	600	0.42 (0.05)	281	0.714
**Gender (%)**[a]					0.449
Male	50.74	307	53.45	155	
Female	49.26	298	46.55	135	
**Pubertal development (%)**[c]					
Pre-pubertal	7.17	43	6.41	18	0.805
Pubertal	7.67	46	6.76	19	
Post-pubertal	85.17	511	86.83	244	
**Household assets score (%)**[f]					0.922
Low	23.66	442	23.36	210	
Medium	76.34	137	76.64	64	
High					0.550
**Caregiver Education (%)**[g]	27.07	157	27.01	74	
Illiterate / Incomplete primary education	25.52	148	21.53	59	
Complete primary education/Incomplete secondary education	41.21	239	45.62	125	
Complete secondary education/Incomplete higher education	6.21	36	5.84	16	
Complete higher education					0.913
**Anthropometric status (%)**[h]	5.67	34	6.3	18	
Malnutrition	71.83	431	69.86	197	
Normal weight	14.33	86	14.54	41	
Overweight	8.17	49	9.22	26	
**Physical activity (%)**[i]					0.166
Insufficiently active	88.43	535	85.17	247	
Physically active	11.57	68	14.83	42	
**Sedentary behaviour (%)**[i]					0.328
Sedentary (time TV ≥ 2h)	60.33	240	56.90	125	
Not Sedentary (time TV < 2h)	39.67	363	43.10	164	
**Bean** [a]					0.562
Regular consumption	28.76	174	26.90	78	
Less frequent consumption	71.24	431	73.10	212	
**Fried Snacks** [a]					0.718
Regular consumption	10.58	64	11.38	33	
Less frequent consumption	89.42	541	88.62	257	
**Processed meats** [a]					0.529
Regular consumption	11.40	69	10.0	29	
Less frequent consumption	88.60	536	90.0	261	
**At least one type of vegetables** [a]					0.172
Regular consumption	11.24	68	8.28	24	
Less frequent consumption	88.76	537	91.72	266	

**Raw salad** [a]					0.158
Regular consumption	17.52	106	13.79	40	
Less frequent consumption	82.48	499	86.21	250	
**Vegetables or legumes in food** [a]					0.943
Regular consumption	16.36	99	16.55	48	
Less frequent consumption	83.64	506	83.45	242	
**Crackers** [a]					0.597
Regular consumption	27.27	165	28.97	84	
Less frequent consumption	72.73	440	71.03	206	
**Cookies** [a]					0.848
Regular consumption	25.95	157	26.55	77	
Less frequent consumption	74.05	448	73.45	213	
**Snacks** [a]					0.558
Regular consumption	11.74	71	13.10	38	
Less frequent consumption	88.26	534	86.90	252	
**Candies** [a]					0.770
Regular consumption	35.21	213	36.21	105	
Less frequent consumption	64.79	392	63.79	185	
**Fruits** [a]					0.293
Regular consumption	19.83	120	16.90	49	
Less frequent consumption	80.17	485	83.10	241	
**Milk** [a]					**0.029**
Regular consumption	38.18	231	30.69	89	
Less frequent consumption	61.82	374	69.31	201	
**Soft drinks** [a]					0.862
Regular consumption	29.09	176	29.66	86	
Less frequent consumption	70.91	429	70.34	204	

μ= mean; a= 605/290; b= 600/281; c= 597/274; d= 580/274; e = 600/2822; f= 453/195; g= 603/289.

Table S2: Loss analyses between participants and dropouts of the study according to groups studied. Salvador. 2017.

Variables	Participants				Dropouts				p
	Interv		Control		Interv		Control		
	%/µ	N	%/µ	N	%/µ	N	%/µ	N	
**Age (in years)**[a] - mean	14.30	285	14.20	320	14.85	143	15.14	147	**0.000**
**Weight (kg)**[b] - mean	55.51	284	53.55	317	57.16	141	55.71	142	**0.045**
**Waist circumference (cm)** [c]- mean	70.25	284	68.45	316	70.53	140	69.53	141	0.064
**Hip circumference (cm)** [d] - mean	88.57	284	87.80	316	89.50	139	89.32	139	0.256
**Arm circumference (cm)** [e] - mean	84.92	284	24.06	316	25.43	141	24.73	139	**0.003**
**Triceps skinfold (mm)** [d] - mean	13.95	282	13.79	315	14.31	141	13.71	140	0.862
**Subscapular skinfold (mm)** [d] - mean	14.71	282	14.41	315	14.72	141	14.69	140	0.949
**Sum of two folds (mm)** [d] - mean	28.57	282	28.19	315	28.92	141	28.48	140	0.955
**Waist-to-height ratio** [c] - mean	0.43	282	0.42	316	0.43	141	0.42	140	0.389
**Gender (%)**[a]									0.476
Male	52.63	150	49.06	157	56.64	81	50.34	74	
Female	47.37	135	50.94	163	43.36	62	49.66	73	
**Pubertal development (%)**[c]									0.976
Pre-pubertal	7.07	20	7.26	23	6.43	9	6.38	9	
Pubertal	6.71	19	8.52	27	6.43	9	7.09	10	
Post-pubertal	86.22	244	84.23	267	86.52	122	87.14	122	
**Household assets score (%)**[f]									0.196
Low	19.55	52	27.16	85	24.03	31	22.76	33	
Medium	80.45	214	72.84	228	75.97	98	77.24	112	
High									0.667
**Caregiver Education (%)**[g]	24.72	66	29.07	91	22.48	29	31.03	45	
Illiterate / Incomplete primary education	26.97	72	24.28	76	22.48	29	20.69	30	
Complete primary education/Incomplete secondary education	41.57	111	40.89	128	49.61	64	42.07	61	
Complete secondary education/Incomplete higher education	6.74	18	5.75	18	5.43	7	6.21	9	
Complete higher education									0.653
**Anthropometric status (%)**[h]	5.99	17	5.38	17	5.67	8	7.09	10	
Malnutrition	68.66	195	74.68	236	72.34	102	67.38	95	
Normal weight	15.49	44	13.29	42	11.35	16	17.73	25	
Overweight	9.86	28	6.65	21	10.64	15	7.8	11	
**Physical activity (%)**[i]									
Insufficiently active	25.12	52	23.58	58	22.77	23	22.34	21	0.945
Physically active	59.90	124	60.16	148	49.50	50	57.45	54	0.286
**Sedentary behaviour (%)**[i]	26.09	54	21.95	54	27.72	28	18.09	17	0.306
Sedentary (time TV ≥ 2h)	23.19	48	16.26	40	33.66	34	22.34	21	**0.005**
Not Sedentary (time TV < 2h)									0.243
**Gender (%)**[a]	89.79	255	87.77	280	88.03	125	82.99	122	
Male	10.21	29	12.23	39	11.97	17	17.01	25	
Female									**0.039**
**Pubertal development (%)**[c]	64.08	182	56.74	181	62.68	89	51.02	75	
Pre-pubertal	35.92	102	43.26	138	37.32	53	48.98	72	
**Bean** [a]									0.316
Regular consumption	26.67	76	30.63	98	23.08	33	30.61	45	
Less frequent consumption	73.33	209	69.38	222	76.92	110	69.39	102	
**Fried Snacks** [a]									0.961
Regular consumption	10.18	256	10.94	35	11.89	17	10.88	16	
Less frequent consumption	89.82	29	89.06	285	88.11	126	89.12	131	
**Processed meats** [a]									0.316
Regular consumption	11.23	32	11.56	37	13.29	19	6.80	10	
Less frequent consumption	88.77	253	88.44	283	86.71	124	93.20	137	
**At least one type of vegetables** [a]									0.463
Regular consumption	11.23	32	11.25	36	9.79	14	6.80	10	

Less frequent consumption	88.77	253	88.75	284	90.21	129	93.20	137	
**Raw salad** [a]									0.505
Regular consumption	16.84	48	18.13	58	14.69	21	12.93	19	
Less frequent consumption	83.16	237	81.88	262	85.31	122	87.07	128	
**Vegetables or legumes in food** [a]									0.510
Regular consumption	14.04	40	18.44	59	17.48	25	15.65	23	
Less frequent consumption	85.96	245	81.56	261	82.52	118	84.35	124	
**Crackers** [a]									0.268
Regular consumption	30.88	88	24.06	77	30.07	43	27.89	41	
Less frequent consumption	69.12	197	75.94	243	69.93	100	72.11	106	
**Cookies** [a]									0.373
Regular consumption	29.12	83	23.13	74	25.17	36	27.89	41	
Less frequent consumption	70.88	202	76.88	246	74.83	107	72.11	106	
**Snacks** [a]									0.476
Regular consumption	12.98	37	10.63	34	15.38	53	10.88	52	
Less frequent consumption	87.02	248	89.38	286	84.62	90	89.12	95	
**Candies** [a]									0.968
Regular consumption	35.79	102	34.69	111	37.06	53	35.37	52	
Less frequent consumption	64.21	183	65.31	209	62.94	90	64.63	95	
**Fruits** [a]									0.701
Regular consumption	19.65	56	20.0	64	18.18	26	15.65	23	
Less frequent consumption	80.35	229	80.0	256	81.02	117	84.35	124	
**Milk** [a]									0.096
Regular consumption	40.70	116	35.94	115	31.47	45	29.93	44	
Less frequent consumption	59.30	169	64.06	205	68.53	98	70.07	103	
**Soft drinks** [a]									0.068
Regular consumption	32.63	93	25.94	83	34.97	50	24.49	36	
Less frequent consumption	65.03	192	75.51	237	67.37	93	74.06	111	

μ= mean; a= 285/320/143/147; b= 283/217/140/141; c=266/313/129/145; d= 267/313/129/145; e= 284/316/141/ 141; f=207/242/101/94 ; g= 284/319/142/ 147.
